# The impact of endoplasmic reticulum stress on neutrophil function in alpha 1 antitrypsin deficiency

**DOI:** 10.1186/1753-6561-9-S1-A4

**Published:** 2015-01-14

**Authors:** M Shaharom, H Kerr, D Bergin, K Hurley

**Affiliations:** 1 Royal College of Surgeons in Ireland, 123 St. Stephen’s Green, Dublin 2. Ireland; 2 Respiratory Research Division, Beaumont Hospital, Dublin 9. Ireland

## Background

Alpha-1 antitrypsin (AAT) deficiency (AATD) is an autosomal recessive disorder characterized by reduced serum AAT levels. This results in little protection for the lower airways against destruction by proteolytic enzymes from neutrophils. The resulting chronic destruction of the lung, or emphysema, typically affects individuals with AATD in middle age and progresses slowly [1]. The ‘Z’ variant is the most common mutation held responsible for >95% of AATD cases. The Z mutation results in misfolding of the AAT protein leading to its accumulation in the endoplasmic reticulum (ER). In monocytes, the consequences of Z-AAT retention within the ER has been shown to activate the unfolded protein response (UPR) leading to proinflammatory cytokine production [2]. As neutrophils are the primary effector cell responsible for the pathological manifestations of AATD lung disease, the aim of this study was to determine whether the UPR occurs in neutrophils as a result of ER stress and the impact this pathway might have on the release proteolytic enzymes from these cells.

## Methods

Neutrophils were isolated from both MM healthy controls and ZZ AATD patients. Cellular levels of ATF-6 (transcription factor) and GRP-78 (chaperone protein) which are markers of ER stress were quantified by Western blot. Purified MM neutrophils were cultured ± thapsigargin (1uM), a chemical inducer of ER stress. Cellular markers of ER stress were measured by Western blot. Degranulation level of MM neutrophils treated ± thapsigargin for 10 mins was determined by Western blot analysis of cell free supernatants using antibodies against MMP-9 (tertiary granules) and hCAP-18 (secondary granules). Statistical analysis was performed using GraphPad Prism 4.0. Ethical approval was obtained from the Beaumont Hospital ethic committee.

## Results

Analysis of ATF-6 and GRP-78 protein expression revealed that ZZ-AATD neutrophils had an increase in the levels of these ER stress markers when compared to healthy control cells (P<0.05). Inducing ER stress in MM cells with thapsigargin also resulted in elevated ATF-6 and GRP-78 protein expression when compared to untreated cells. Furthermore, the treatment of MM neutrophils with thapsigargin resulted in a 10-fold and 4-fold increase in the release of MMP-9 and hCAP-18 respectively when compared to untreated MM-cells.

## Conclusions

In conclusion, we document for the first time ER stress and the UPR response in ZZ-AATD neutrophils. Furthermore we demonstrate that ER stress and UPR can increase neutrophil degranulation. This shifts the paradigm of inflammation in AATD beyond lung and liver cells to include circulating immune cells.
